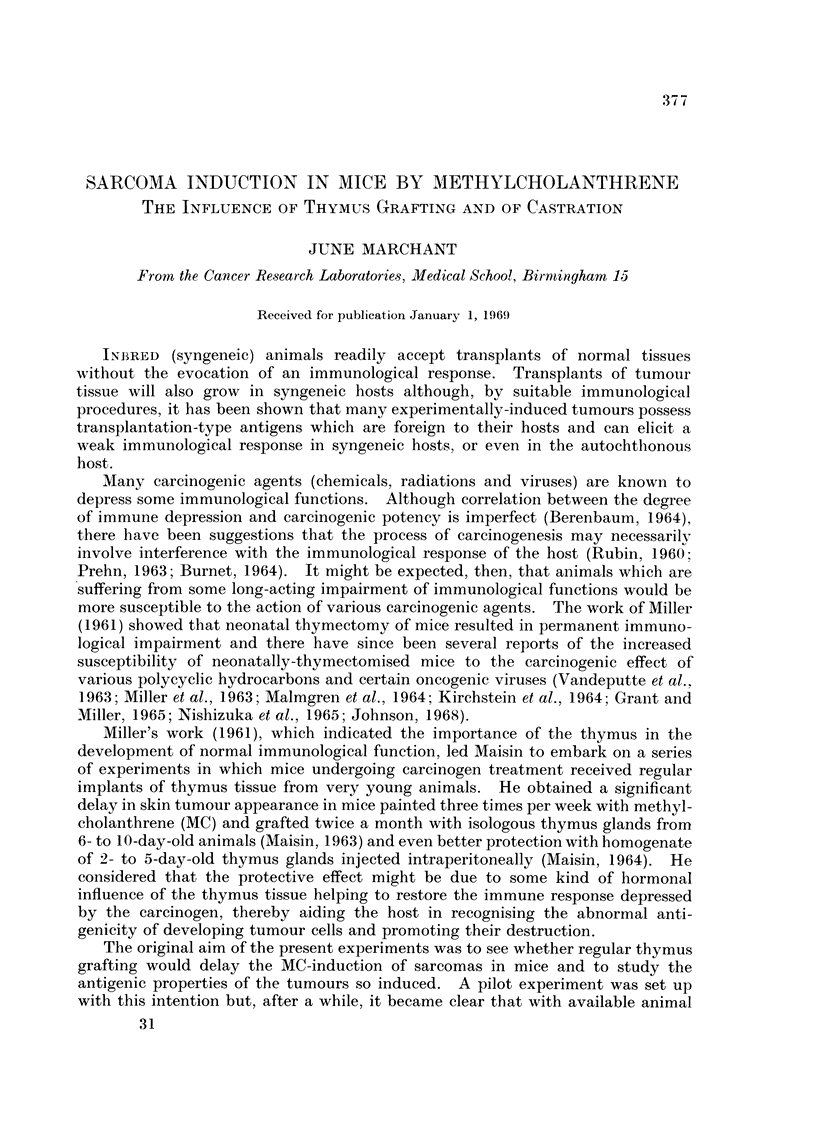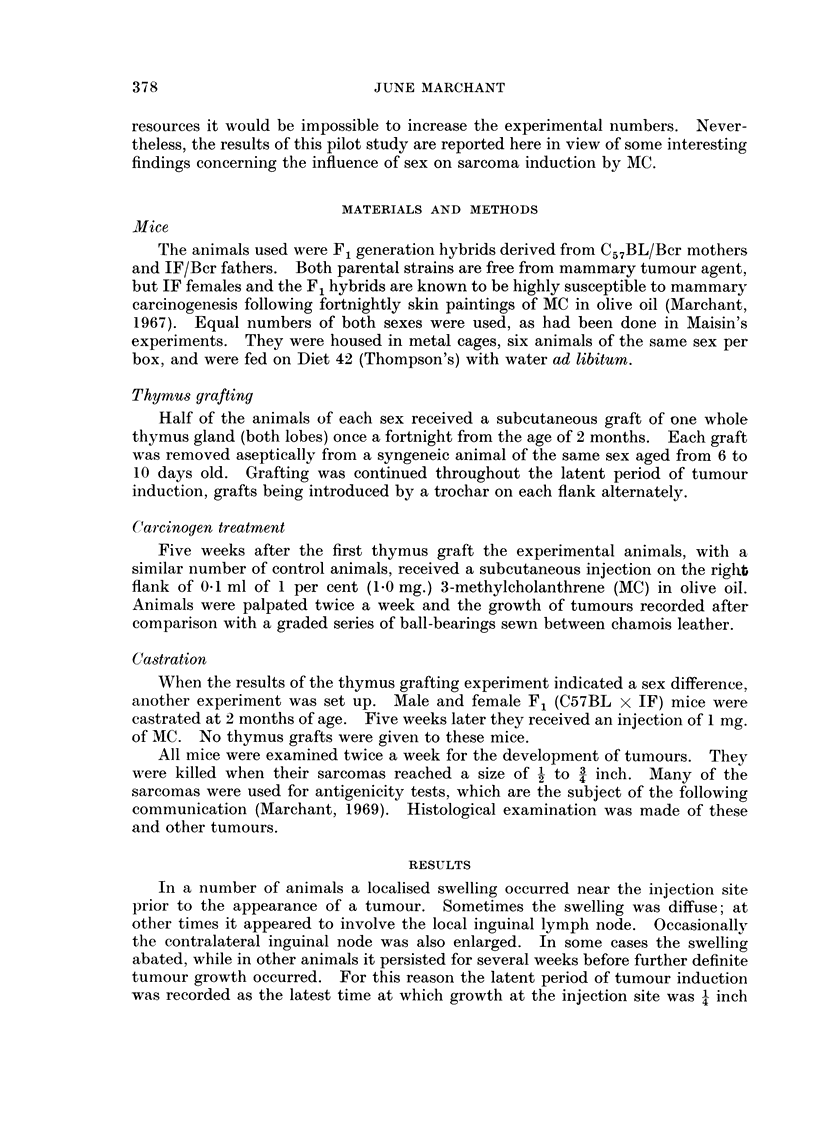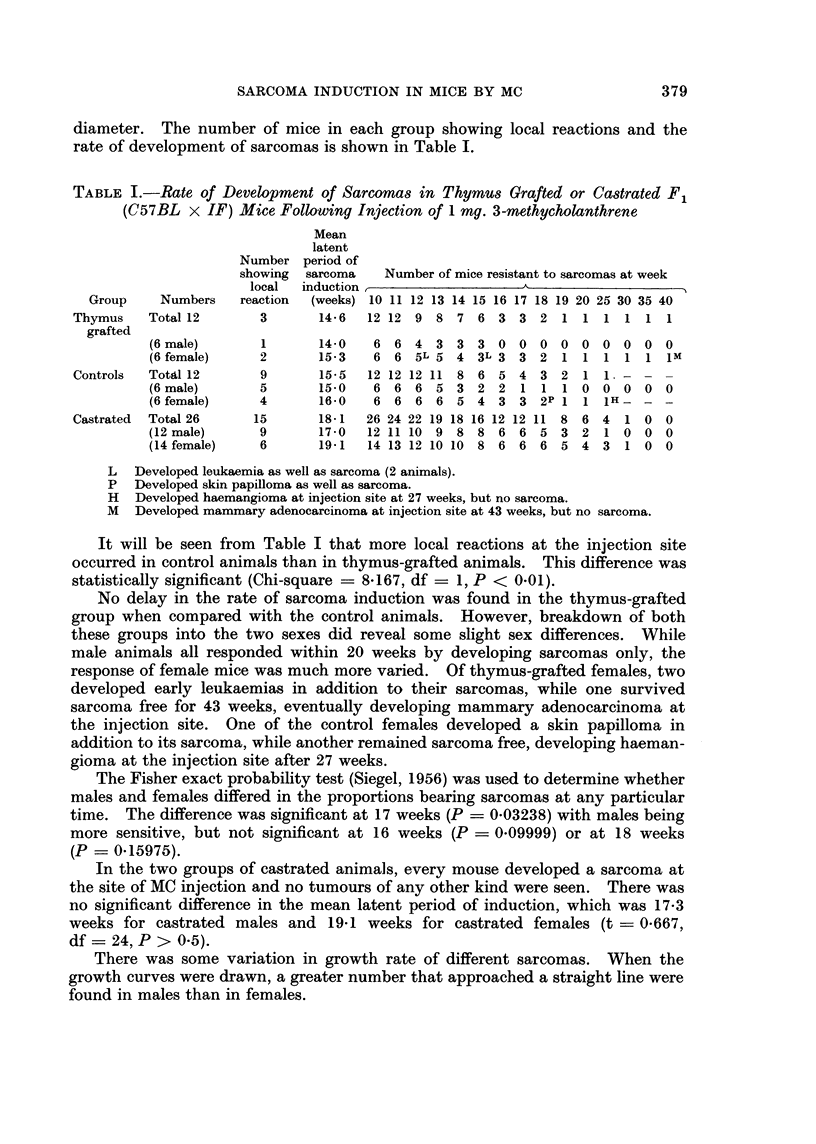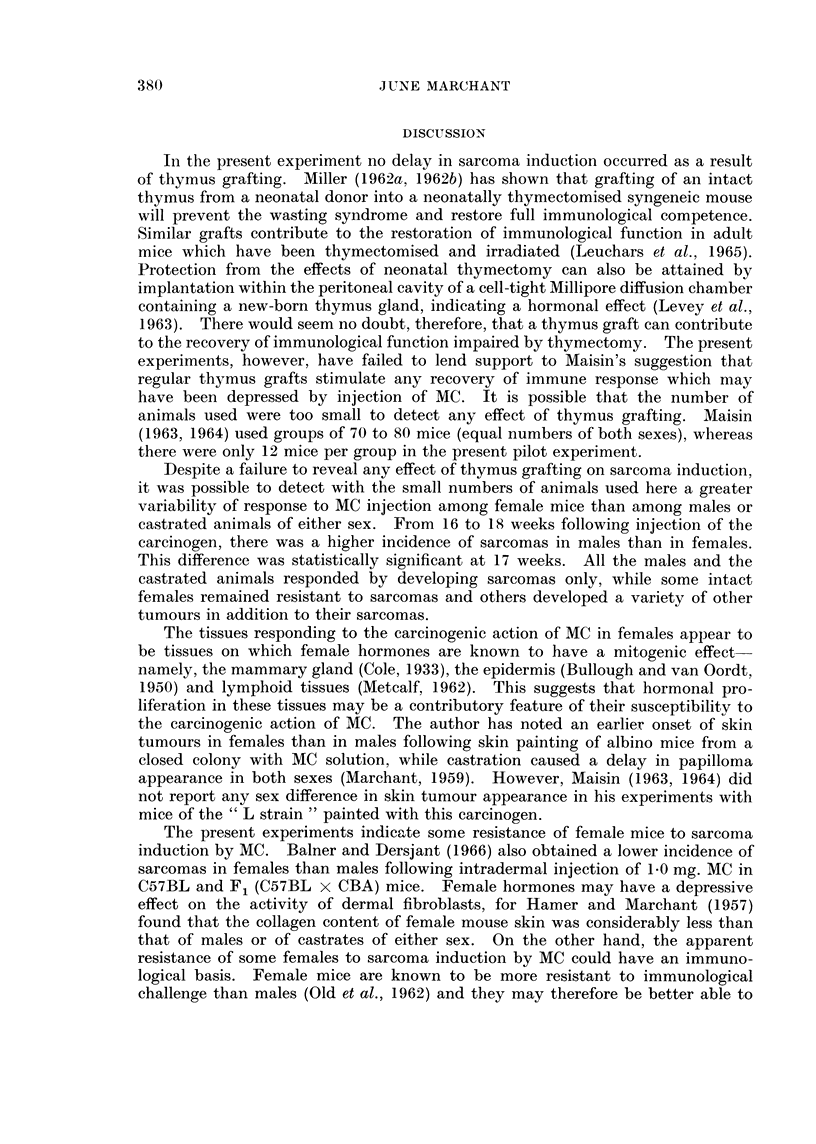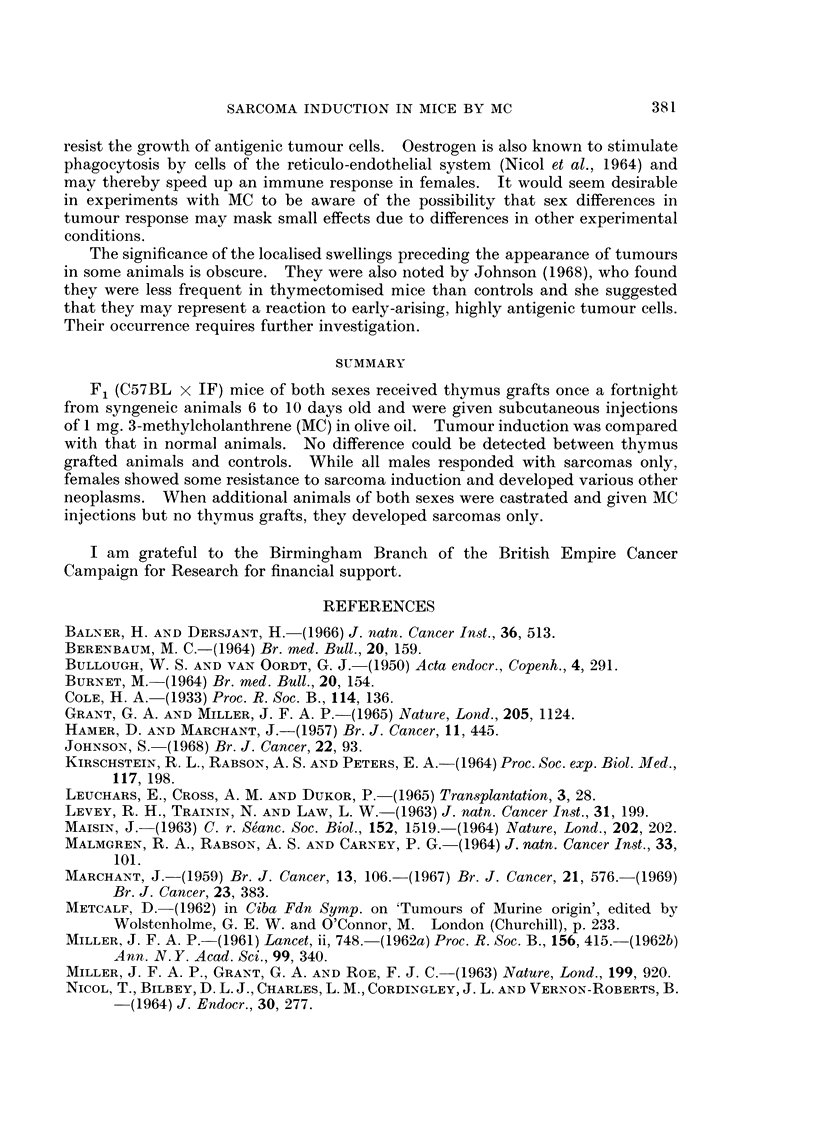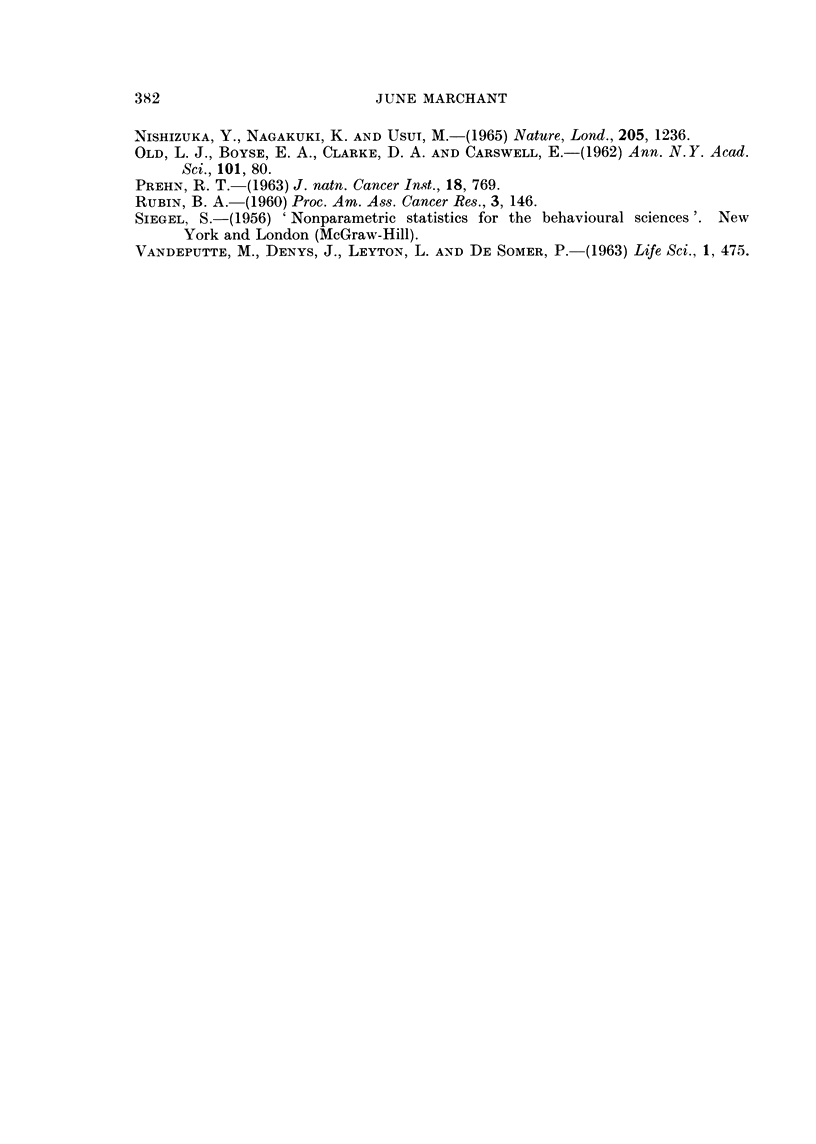# Sarcoma induction in mice by methylcholanthrene. The influence of thymus grafting and of castration.

**DOI:** 10.1038/bjc.1969.49

**Published:** 1969-06

**Authors:** J. Marchant


### SARCOMA INDUCTION IN MICE BY METHYLCHOLANTHRENE

### The Influence of Thymus Grafting and of Castration

JUNE MARCHANT

*From the Cancer Research Laboratories, Medical School, Birmingham 15*

Received for publication January 1, 1969

INBRED (syngeneic) animals readily accept transplants of normal tissues without the evocation of an immunological response. Transplants of tumour tissue will also grow in syngeneic hosts although, by suitable immunological procedures, it has been shown that many experimentally-induced tumours possess transplantation-type antigens which are foreign to their hosts and can elicit a weak immunological response in syngeneic hosts, or even in the autochthonous host.

Many carcinogenic agents (chemicals, radiations and viruses) are known to depress some immunological functions. Although correlation between the degree of immune depression and carcinogenic potency is imperfect (Berenbaum, 1964), there have been suggestions that the process of carcinogenesis may necessarily involve interference with the immunological response of the host (Rubin, 1960; Prehn, 1963; Burnet, 1964). It might be expected, then, that animals which are suffering from some long-acting impairment of immunological functions would be more susceptible to the action of various carcinogenic agents. The work of Miller (1961) showed that neonatal thymectomy of mice resulted in permanent immunological impairment and there have since been several reports of the increased susceptibility of neonatally-thymectomised mice to the carcinogenic effect of various polycyclic hydrocarbons and certain oncogenic viruses (Vandeputte *et al.*, 1963; Miller *et al.*, 1963; Malmgren *et al.*, 1964; Kirchstein *et al.*, 1964; Grant and Miller, 1965; Nishizuka *et al.*, 1965; Johnson, 1968).

Miller's work (1961), which indicated the importance of the thymus in the development of normal immunological function, led Maisin to embark on a series of experiments in which mice undergoing carcinogen treatment received regular implants of thymus tissue from very young animals. He obtained a significant delay in skin tumour appearance in mice painted three times per week with methylcholanthrene (MC) and grafted twice a month with isologous thymus glands from 6- to 10-day-old animals (Maisin, 1963) and even better protection with homogenate of 2- to 5-day-old thymus glands injected intraperitoneally (Maisin, 1964). He considered that the protective effect might be due to some kind of hormonal influence of the thymus tissue helping to restore the immune response depressed by the carcinogen, thereby aiding the host in recognising the abnormal antigenicity of developing tumour cells and promoting their destruction.

The original aim of the present experiments was to see whether regular thymus grafting would delay the MC-induction of sarcomas in mice and to study the antigenic properties of the tumours so induced. A pilot experiment was set up with this intention but, after a while, it became clear that with available animal resources it would be impossible to increase the experimental numbers. Nevertheless, the results of this pilot study are reported here in view of some interesting findings concerning the influence of sex on sarcoma induction by MC.

### MATERIALS AND METHODS

### *Mice*

The animals used were $F_1$ generation hybrids derived from $C_{57}BL/Bcr$ mothers and IF/Bcr fathers. Both parental strains are free from mammary tumour agent, but IF females and the $F_1$ hybrids are known to be highly susceptible to mammary carcinogenesis following fortnightly skin paintings of MC in olive oil (Marchant, 1967). Equal numbers of both sexes were used, as had been done in Maisin's experiments. They were housed in metal cages, six animals of the same sex per box, and were fed on Diet 42 (Thompson's) with water *ad libitum*.

### *Thymus grafting*

Half of the animals of each sex received a subcutaneous graft of one whole thymus gland (both lobes) once a fortnight from the age of 2 months. Each graft was removed aseptically from a syngeneic animal of the same sex aged from 6 to 10 days old. Grafting was continued throughout the latent period of tumour induction, grafts being introduced by a trochar on each flank alternately.

### *Carcinogen treatment*

Five weeks after the first thymus graft the experimental animals, with a similar number of control animals, received a subcutaneous injection on the right flank of 0·1 ml of 1 per cent (1·0 mg.) 3-methylcholanthrene (MC) in olive oil. Animals were palpated twice a week and the growth of tumours recorded after comparison with a graded series of ball-bearings sewn between chamois leather.

### *Castration*

When the results of the thymus grafting experiment indicated a sex difference, another experiment was set up. Male and female $F_1$ (C57BL $\times$ IF) mice were castrated at 2 months of age. Five weeks later they received an injection of 1 mg. of MC. No thymus grafts were given to these mice.

All mice were examined twice a week for the development of tumours. They were killed when their sarcomas reached a size of $\frac{1}{2}$ to $\frac{3}{4}$ inch. Many of the sarcomas were used for antigenicity tests, which are the subject of the following communication (Marchant, 1969). Histological examination was made of these and other tumours.

### RESULTS

In a number of animals a localised swelling occurred near the injection site prior to the appearance of a tumour. Sometimes the swelling was diffuse; at other times it appeared to involve the local inguinal lymph node. Occasionally the contralateral inguinal node was also enlarged. In some cases the swelling abated, while in other animals it persisted for several weeks before further definite tumour growth occurred. For this reason the latent period of tumour induction was recorded as the latest time at which growth at the injection site was $\frac{1}{4}$ inch diameter. The number of mice in each group showing local reactions and the rate of development of sarcomas is shown in Table I.

TABLE I.—*Rate of Development of Sarcomas in Thymus Grafted or Castrated* $F_1$ *(C57BL × IF) Mice Following Injection of* 1 *mg.* 3-*methycholanthrene*

| Group | Numbers | Number showing local reaction | Mean latent period of sarcoma induction (weeks) | Number of mice resistant to sarcomas at week | | | | | | | | | | | | | | |
|---|---|---|---|---|---|---|---|---|---|---|---|---|---|---|---|---|---|---|
| | | | | 10 | 11 | 12 | 13 | 14 | 15 | 16 | 17 | 18 | 19 | 20 | 25 | 30 | 35 | 40 |
| Thymus grafted | Total 12 | 3 | 14·6 | 12 | 12 | 9 | 8 | 7 | 6 | 3 | 3 | 2 | 1 | 1 | 1 | 1 | 1 | 1 |
| | (6 male) | 1 | 14·0 | 6 | 6 | 4 | 3 | 3 | 3 | 0 | 0 | 0 | 0 | 0 | 0 | 0 | 0 | 0 |
| | (6 female) | 2 | 15·3 | 6 | 6 | 5[L] | 5 | 4 | 3[L] | 3 | 3 | 2 | 1 | 1 | 1 | 1 | 1 | 1[M] |
| Controls | Total 12 | 9 | 15·5 | 12 | 12 | 12 | 11 | 8 | 6 | 5 | 4 | 3 | 2 | 1 | 1 | – | – | – |
| | (6 male) | 5 | 15·0 | 6 | 6 | 6 | 5 | 3 | 2 | 2 | 1 | 1 | 1 | 0 | 0 | 0 | 0 | 0 |
| | (6 female) | 4 | 16·0 | 6 | 6 | 6 | 6 | 5 | 4 | 3 | 3 | 2[P] | 1 | 1 | 1[H] | – | – | – |
| Castrated | Total 26 | 15 | 18·1 | 26 | 24 | 22 | 19 | 18 | 16 | 12 | 12 | 11 | 8 | 6 | 4 | 1 | 0 | 0 |
| | (12 male) | 9 | 17·0 | 12 | 11 | 10 | 9 | 8 | 8 | 6 | 6 | 5 | 3 | 2 | 1 | 0 | 0 | 0 |
| | (14 female) | 6 | 19·1 | 14 | 13 | 12 | 10 | 10 | 8 | 6 | 6 | 6 | 5 | 4 | 3 | 1 | 0 | 0 |

L Developed leukaemia as well as sarcoma (2 animals).
P Developed skin papilloma as well as sarcoma.
H Developed haemangioma at injection site at 27 weeks, but no sarcoma.
M Developed mammary adenocarcinoma at injection site at 43 weeks, but no sarcoma.

It will be seen from Table I that more local reactions at the injection site occurred in control animals than in thymus-grafted animals. This difference was statistically significant (Chi-square $= 8{\cdot}167$, df $= 1, P < 0{\cdot}01$).

No delay in the rate of sarcoma induction was found in the thymus-grafted group when compared with the control animals. However, breakdown of both these groups into the two sexes did reveal some slight sex differences. While male animals all responded within 20 weeks by developing sarcomas only, the response of female mice was much more varied. Of thymus-grafted females, two developed early leukaemias in addition to their sarcomas, while one survived sarcoma free for 43 weeks, eventually developing mammary adenocarcinoma at the injection site. One of the control females developed a skin papilloma in addition to its sarcoma, while another remained sarcoma free, developing haemangioma at the injection site after 27 weeks.

The Fisher exact probability test (Siegel, 1956) was used to determine whether males and females differed in the proportions bearing sarcomas at any particular time. The difference was significant at 17 weeks ($P = 0{\cdot}03238$) with males being more sensitive, but not significant at 16 weeks ($P = 0{\cdot}09999$) or at 18 weeks ($P = 0{\cdot}15975$).

In the two groups of castrated animals, every mouse developed a sarcoma at the site of MC injection and no tumours of any other kind were seen. There was no significant difference in the mean latent period of induction, which was 17·3 weeks for castrated males and 19·1 weeks for castrated females ($t = 0{\cdot}667$, df $= 24, P > 0{\cdot}5$).

There was some variation in growth rate of different sarcomas. When the growth curves were drawn, a greater number that approached a straight line were found in males than in females.

### DISCUSSION

In the present experiment no delay in sarcoma induction occurred as a result of thymus grafting. Miller (1962*a*, 1962*b*) has shown that grafting of an intact thymus from a neonatal donor into a neonatally thymectomised syngeneic mouse will prevent the wasting syndrome and restore full immunological competence. Similar grafts contribute to the restoration of immunological function in adult mice which have been thymectomised and irradiated (Leuchars *et al.*, 1965). Protection from the effects of neonatal thymectomy can also be attained by implantation within the peritoneal cavity of a cell-tight Millipore diffusion chamber containing a new-born thymus gland, indicating a hormonal effect (Levey *et al.*, 1963). There would seem no doubt, therefore, that a thymus graft can contribute to the recovery of immunological function impaired by thymectomy. The present experiments, however, have failed to lend support to Maisin's suggestion that regular thymus grafts stimulate any recovery of immune response which may have been depressed by injection of MC. It is possible that the number of animals used were too small to detect any effect of thymus grafting. Maisin (1963, 1964) used groups of 70 to 80 mice (equal numbers of both sexes), whereas there were only 12 mice per group in the present pilot experiment.

Despite a failure to reveal any effect of thymus grafting on sarcoma induction, it was possible to detect with the small numbers of animals used here a greater variability of response to MC injection among female mice than among males or castrated animals of either sex. From 16 to 18 weeks following injection of the carcinogen, there was a higher incidence of sarcomas in males than in females. This difference was statistically significant at 17 weeks. All the males and the castrated animals responded by developing sarcomas only, while some intact females remained resistant to sarcomas and others developed a variety of other tumours in addition to their sarcomas.

The tissues responding to the carcinogenic action of MC in females appear to be tissues on which female hormones are known to have a mitogenic effect—namely, the mammary gland (Cole, 1933), the epidermis (Bullough and van Oordt, 1950) and lymphoid tissues (Metcalf, 1962). This suggests that hormonal proliferation in these tissues may be a contributory feature of their susceptibility to the carcinogenic action of MC. The author has noted an earlier onset of skin tumours in females than in males following skin painting of albino mice from a closed colony with MC solution, while castration caused a delay in papilloma appearance in both sexes (Marchant, 1959). However, Maisin (1963, 1964) did not report any sex difference in skin tumour appearance in his experiments with mice of the " L strain " painted with this carcinogen.

The present experiments indicate some resistance of female mice to sarcoma induction by MC. Balner and Dersjant (1966) also obtained a lower incidence of sarcomas in females than males following intradermal injection of 1·0 mg. MC in C57BL and $F_1$ (C57BL $\times$ CBA) mice. Female hormones may have a depressive effect on the activity of dermal fibroblasts, for Hamer and Marchant (1957) found that the collagen content of female mouse skin was considerably less than that of males or of castrates of either sex. On the other hand, the apparent resistance of some females to sarcoma induction by MC could have an immunological basis. Female mice are known to be more resistant to immunological challenge than males (Old *et al.*, 1962) and they may therefore be better able to resist the growth of antigenic tumour cells. Oestrogen is also known to stimulate phagocytosis by cells of the reticulo-endothelial system (Nicol *et al.*, 1964) and may thereby speed up an immune response in females. It would seem desirable in experiments with MC to be aware of the possibility that sex differences in tumour response may mask small effects due to differences in other experimental conditions.

The significance of the localised swellings preceding the appearance of tumours in some animals is obscure. They were also noted by Johnson (1968), who found they were less frequent in thymectomised mice than controls and she suggested that they may represent a reaction to early-arising, highly antigenic tumour cells. Their occurrence requires further investigation.

SUMMARY

$F_1$ (C57BL $\times$ IF) mice of both sexes received thymus grafts once a fortnight from syngeneic animals 6 to 10 days old and were given subcutaneous injections of 1 mg. 3-methylcholanthrene (MC) in olive oil. Tumour induction was compared with that in normal animals. No difference could be detected between thymus grafted animals and controls. While all males responded with sarcomas only, females showed some resistance to sarcoma induction and developed various other neoplasms. When additional animals of both sexes were castrated and given MC injections but no thymus grafts, they developed sarcomas only.

I am grateful to the Birmingham Branch of the British Empire Cancer Campaign for Research for financial support.

### REFERENCES

Balner, H. and Dersjant, H.—(1966) *J. natn. Cancer Inst.*, **36**, 513.
Berenbaum, M. C.—(1964) *Br. med. Bull.*, **20**, 159.
Bullough, W. S. and van Oordt, G. J.—(1950) *Acta endocr., Copenh.*, **4**, 291.
Burnet, M.—(1964) *Br. med. Bull.*, **20**, 154.
Cole, H. A.—(1933) *Proc. R. Soc.* B., **114**, 136.
Grant, G. A. and Miller, J. F. A. P.—(1965) *Nature, Lond.*, **205**, 1124.
Hamer, D. and Marchant, J.—(1957) *Br. J. Cancer*, **11**, 445.
Johnson, S.—(1968) *Br. J. Cancer*, **22**, 93.
Kirschstein, R. L., Rabson, A. S. and Peters, E. A.—(1964) *Proc. Soc. exp. Biol. Med.*, **117**, 198.
Leuchars, E., Cross, A. M. and Dukor, P.—(1965) *Transplantation*, **3**, 28.
Levey, R. H., Trainin, N. and Law, L. W.—(1963) *J. natn. Cancer Inst.*, **31**, 199.
Maisin, J.—(1963) *C. r. Séanc. Soc. Biol.*, **152**, 1519.—(1964) *Nature, Lond.*, **202**, 202.
Malmgren, R. A., Rabson, A. S. and Carney, P. G.—(1964) *J. natn. Cancer Inst.*, **33**, 101.
Marchant, J.—(1959) *Br. J. Cancer*, **13**, 106.—(1967) *Br. J. Cancer*, **21**, 576.—(1969) *Br. J. Cancer*, **23**, 383.
Metcalf, D.—(1962) in *Ciba Fdn Symp.* on 'Tumours of Murine origin', edited by Wolstenholme, G. E. W. and O'Connor, M. London (Churchill), p. 233.
Miller, J. F. A. P.—(1961) *Lancet*, ii, 748.—(1962*a*) *Proc. R. Soc.* B., **156**, 415.—(1962*b*) *Ann. N.Y. Acad. Sci.*, **99**, 340.
Miller, J. F. A. P., Grant, G. A. and Roe, F. J. C.—(1963) *Nature, Lond.*, **199**, 920.
Nicol, T., Bilbey, D. L. J., Charles, L. M., Cordingley, J. L. and Vernon-Roberts, B. —(1964) *J. Endocr.*, **30**, 277.

NISHIZUKA, Y., NAGAKUKI, K. AND USUI, M.—(1965) *Nature, Lond.*, **205**, 1236.

OLD, L. J., BOYSE, E. A., CLARKE, D. A. AND CARSWELL, E.—(1962) *Ann. N.Y. Acad. Sci.*, **101**, 80.

PREHN, R. T.—(1963) *J. natn. Cancer Inst.*, **18**, 769.

RUBIN, B. A.—(1960) *Proc. Am. Ass. Cancer Res.*, **3**, 146.

SIEGEL, S.—(1956) 'Nonparametric statistics for the behavioural sciences'. New York and London (McGraw-Hill).

VANDEPUTTE, M., DENYS, J., LEYTON, L. AND DE SOMER, P.—(1963) *Life Sci.*, **1**, 475.